# Doctor can I buy a new kidney? I've heard it isn't forbidden: what is the role of the nephrologist when dealing with a patient who wants to buy a kidney?

**DOI:** 10.1186/s13010-015-0033-x

**Published:** 2015-12-18

**Authors:** Giorgina Barbara Piccoli, Laura Sacchetti, Laura Verzè, Franco Cavallo, Working group of the students of the Torino Medical School

**Affiliations:** SS Nephrology, Department of Clinical and Biological Sciences University of Torino, Torino, Italy; AOU san Luigi Gonzaga, Regione Gonzole 10, Orbassano, Torino, Italy; EBM Course, Torino Medical School, University of Torino, Torino, Italy

## Abstract

Organ trafficking is officially banned in several countries and by the main Nephrology Societies. However, this practice is widespread and is allowed or tolerated in many countries, hence, in the absence of a universal law, the caregiver may be asked for advice, placing him/her in a difficult balance between legal aspects, moral principles and ethical judgments.

In spite of the Istanbul declaration, which is a widely shared position statement against organ trafficking, the controversy on mercenary organ donation is still open and some experts argue against taking a negative stance. In the absence of clear evidence showing the clinical disadvantages of mercenary transplantation compared to chronic dialysis, self-determination of the patient (and, with several caveats, of the donor) may conflict with other ethical principles, first of all non-maleficence. The present paper was drawn up with the participation of the students, as part of the ethics course at our medical school. It discusses the situation in which the physician acts as a counselor for the patient in the way of a sort of “reverse” informed consent, in which the patient asks advice regarding a complex personal decision, and includes a peculiar application of the four principles (beneficence, non-maleficence, justice and autonomy) to the donor and recipient parties.

## Introduction

Almost 30 years ago, an Editorial in The Lancet entitled “Who owns Medical Technology” told a fascinating story of Lapps, reindeer and snowmobiles, and commented that technology brings much more than hardware: it may lead to a true shift in social values [1]. Indeed, new technologies bring new ethical problems and the issue of transplantation is probably one of the best examples involving several aspects of life, not only in Medicine: suffice it to mention the “new definition” of death, i.e. brain-death, that is at the basis of cadaveric organ donation [2–4].

The roots of bioethics are deeply planted in the history of dialysis and of renal replacement therapy: the limited availability of dialysis laid the foundation for the first Ethics Committee, in Seattle, aimed at supporting the decision of allocating dialysis treatment to a few patients, among several potential candidates [5, 6]. The limited availability of of kidneys from cadaveric donors currently poses similar problems in several settings where dialysis is available without restrictions; the clinical and ethical complexity is greater in limited resources settings in which a kidney transplant is often the only therapy potentially leading to long-term survival [5–7].

Globalization, both from a financial and from a cultural point of view, puts people in touch [8]. This sets the stage for the trade of human organs and for transplant tourism, which is on the rise, thus raising extensive and complex clinical and ethical controversy. According to a recent review on stressful ethical issues in uremia therapy, “the voluntary sale of purchased donor kidneys now accounts for thousands of black market transplants amounting to an estimated one-quarter of all kidney transplants performed globally” [7].

Even though many eminent medical associations condemn the sale of human organs, and several religious authorities, including Pope John Paul II, have openly spoken against organ trade because it violates ‘the dignity of the human person’, the problems posed by organ shortages continue to increase in rich as well as in poor countries, almost ironically in parallel with the improvements in kidney transplantation and with the consequent broadening of the indications, in turn increasing the number of potential recipients [7, 9–10, 11–14]. The need for incentives in favor of organ donation is evident although the modality is a matter of controversy [15, 16].

While medical associations and individual nations strive to find common objectives, the problem of kidneys for sale merges into the universal issues of poverty and exploitation [7, 17]. The current technology allows for the broader use and trade of body parts, including “wombs for rent” by surrogate mothers, which is legal in some countries. However, it also poses problems which are often, at least partly, shared by the kidney trade [18–20].

Opponents to the ban on kidney vending object that “paid organ donors need not be victims who have not lost the right to determine what happens to their body”; similar objections are posed in the case of surrogate mothers [7, 15–17, 21].

As will be discussed later, the marketing of human organs is not always illegal, and some countries actually allow the purchase of kidneys, while in other settings, the lack of legislation automatically becomes synonymous of permission [21–27].

In this ever changing, complex and tumultuous scenario, patients with severe chronic kidney disease may have access to various “offers” via the Internet (a famous case occurred a few years ago on e-bay), and may even be contacted by “brokers” [28–30].

The case discussed herein is based on a patient who was followed up in our clinical practice. His story was modified in order to respect the privacy of the patient and of his family. This case was selected for discussion within the course of medical ethics and EBM at the Medical School of the University of Torino, Italy. The present report summarizes the work that was done with the students who were tutored by a nephrologist and two bioethicists.

## Case history

A 65 year old man with chronic kidney disease stage 4 (GFR 20 mL/min) sought medical advice, asking his caregiver nephrologist: “Doctor can I buy a new kidney? I've heard it isn't prohibited in other Countries, and in any case, I’m willing to pay as much as needed to get a new life.”

The patient’s history was unremarkable. His chronic kidney disease was presumably on a vascular-atherosclerotic basis. He had undergone stenting of a renal artery 7 years earlier. After the procedure, his blood pressure reportedly normalized and the patient dropped out of nephrological follow-up. One and a half years before the present discussion, he was referred to the nephrologist after being taken to the Emergency Room following a car accident, where high serum creatinine was found (3.3 mg/dL) in the context of severe hypertension (200/115 mmHg). At that time the patient was not taking any medication. No evidence of recurring renal artery stenosis was found and the patient was started on ACE-inhibitors and Calcium antagonists for blood pressure control, antiuricemic agents, vitamin D, oral bicarbonate, EPO therapy and a low protein diet on account of the need to correct the associated metabolic derangements.

After a phase of reactive depression, the patient continued his job as the owner and manager of a medium sized company, and maintained his traveling and social habits, adapting to the low-protein diet intelligently and flexibly. His family, consisting of his wife, a 20-year-old daughter living with the family and attending the local university, and two older sons, one of whom works with his father, supported him in the adaptation to the lifestyle changes and helped him overcome the initial opposition to “having to take too many pills”. The issue of living donor transplantation had been discussed within the family, however his wife was ABO incompatible and border-line hypertensive, while the patient himself refused the idea of accepting a kidney from one of his children. Despite good compliance to both his diet and drug therapy, the kidney disease progressed and the patient started undergoing clinical and imaging evaluations for wait-listing for a cadaveric kidney graft.

The patient asked his caregiver nephrologist for advice.

### The physician as a counselor: “reverse” informed consent

The patient’s request had some peculiar characteristics: in fact, it does not deal with the choice of a specific treatment (kidney transplantation), which had been agreed was potentially the most favorable choice, nor did it deal with the idea of a related (children) or unrelated (wife) living donation (once more it was agreed to be potentially useful), but with its legal, moral and ethical aspects.

Answering the patient’s request does not imply having technical knowledge (transplantation versus dialysis, living related, or unrelated transplantation), but it does require knowledge of the social and ethical aspects of Medicine (which are not a usual part of medical expertise) as well as of the usual clinical work-up.

Furthermore, answering the patient’s request requires that the physician clearly define his/her role:/ i.e., a counselor with a “parental” role, a partner in a shared decision, or a technical expert who may refuse to answer ethical questions that are not strictly related to his/her work and expertise [31–33].

Each of these roles may be ethically and clinically sound. However, the answers may be substantially different depending on these behavioral models of patient/physician interaction: the parental, or paternalistic counselor would try to convince the patient that his choice bears severe ethical problems and would underline the complex relationship between moral, legal and ethical issues, notwithstanding the clinical concerns. The partner in a shared decision would act as a friend, who understands, listens and participates, and who gives his opinion without attributing a value of “right or justice”. The technical expert would explain the clinical risks, and limit any additional indications to suggestions that might further unravel the ethical aspects, possibly with the help of a specialist. While the physician who follows an exclusively technical approach may somehow avoid the specific questions by identifying a different “technical expert” to whom the patient can be referred, those who follow a parental model or who pursue a therapeutic alliance should go one step forward in the case analysis and engage in an in-depth discussion with the patient.

### The “double” application of the four principles

The four principles of the so-called principlist ethics offer a useful tool for the analysis of complex ethical problems, while integration with a more flexible narrative approach may be of use in refining pragmatic strategies tailored to the individual cases [34, 35].

In this case the analysis according to the four principles (beneficence, non maleficence, justice and autonomy) is peculiar as it deals with two individuals, the donor and the recipient, and the benefit of one may be in contrast with the harm of the other. Furthermore, the concept of justice may take on different meanings since legal justice, moral justice and ethical justice often partially, but incompletely, overlap.

The definition of the principle of autonomy is likewise complex, not only because it involves two different choices, donating or selling, and receiving or buying, but also because it implies a reflection on the definition (and existence) of autonomy in the context of poverty, as clearly stated in the first principle of the Nuremberg code “The voluntary consent of the human subject is absolutely essential [36]. This means that the person involved should have legal capacity to give consent; should be so situated as to be able to exercise free power of choice, without the intervention of any element of force, fraud, deceit, duress, over-reaching, or other ulterior form of constraint or coercion; and should have sufficient knowledge and comprehension of the elements of the subject matter involved as to enable him/her to make an understanding and enlightened decision” [36–38].

Whether poverty is a form of constraint or coercion is one of the central issues in the discussion on human organ trade [39–42].

### Beneficence

#### First actor: The recipient – the buyer

The expected benefit for the patient affected by severe chronic kidney disease is clear: a longer, better quality life. From a statistical point of view, the patient has a high probability of increasing his/her life expectancy and of improving the quality by being treated by transplantation rather than by dialysis [43–45].

In the context of transplantation, provided that the fundamental clinical requirements are met, living donor transplantation allows better organ and patient survival than deceased donor transplantation [46, 47]. Dialysis vintage is negatively correlated with survival after transplantation, thus leading some Authors suggest that kidney transplant should be performed as early as possible [48, 49].

However, the picture is probably more complex, and there are at least five points that should be discussed with the patient.

First: a statistical benefit in a population is not necessarily a clinical benefit for each patient: the fact that the results of kidney transplantation are better than those of dialysis is not synonymous of success for “our” patient, who should be aware of the limits of his/her clinical decision (for instance, early and late loss of kidney function, increased risk of infection in the short term and possibly of neoplasia in the long term).

Secondly, there is very little data on the long-term outcome of kidney transplants from sold kidneys. Within the limits of the scarce research, several added risks have been reported, including surgical problems and severe infections [50–55].

Thirdly, there are no published data comparing mortality and morbidity on dialysis versus paid organ transplantation.

Fourthly, the main survival comparisons between dialysis and transplantation did not consider the intensive dialysis sessions that may have an added value at least in allowing a “safer wait” for a kidney transplant [56–58].

In conclusion, the advantages the patients assume they will reap are at least partly true when we compare living donor transplantation to dialysis. The perceived advantages are probably over-estimated since the risks for “tourist” transplants may be higher than transplants obtained from non-mercenary donors. However, the lack of information on the long-term results makes this statement only putative. Furthermore, since the patient considers the possibility of what could be considered a “non-conventional” transplant, the advantages of non-conventional dialysis, albeit more intrusive in daily life, should also be taken into consideration.

### Second actor: The donor – the seller

No doubt: the only advantage for the donor is financial.

It is also well known that the donor receives only the crumbs of the total fee paid by the recipient, the vast majority of which goes towards honoraria and hospital fees. The extent of the financial advantage for the donor, or, in other terms, the degree of exploitation, varies greatly around the world, with the fewest benefits and the greatest risks in settings without any regulations as compared to the few settings where strict regulations regarding the sale of organs are in place [59–61].

A recent, large survey from Pakistan, one of the countries regulating the sale of kidneys, highlights that besides the negative effects on the donor’s health, even though the donations had mainly been driven by the need to pay for debts, only a minority of sellers were still financially independent a few years after donation [60].

### Non-maleficence

#### First actor: The recipient – the buyer

As already reported, there is an increased risk associated with “tourist” transplantation, mainly due to an increased risk of infectious diseases and of surgical complications. The risks persist in the medium term, when the patient is back home, and they are enhanced by the fact that quite often the clinical records are not supplied and the communication between the team that will take over chronic care of the patient and the team that performed the kidney transplantation is minimal, if there is any at all [51–55, 62].

There seem to be some great differences among centers, and the statement that the quality of a mercenary transplantation is always low may actually represent an oversimplification; however, it should also be acknowledged that the risks are only partially known [62].

Once more, the extent of the counseling that should be provided merges into the discussion about the role of the caregiver, and perhaps also about one's personal position regarding the marketing of human organs.

Ethical and psychological issues should profoundly matter, however, in our Medline search we did not find any studies regarding the long-term psychological effects of having bought a kidney. Once more, this issue may not be simple and the equation “buyer = a person without ethical concerns” may be oversimplified, and cases like those involving our patient, who is a “good, normal person” integrated in his society and with a satisfactory family life, are far form rare.

In this type of situation, in which the buyer cannot be described as a sinless shark, we may expect the long-term integration of the “new kidney” to be more difficult than previously thought [63–71]. This issue probably also has important cultural differences, taking into account the perspectives of recipients in the Mediterranean area as compared to Anglo-Saxon countries, and on the basis of religious beliefs [63–74].

#### Second “actor”: The donor – the seller

The literature on kidney donors “for financial reasons” is relatively scant and mainly derives from countries such as Pakistan or Iran in which the pragmatic clinical and legal position is in favor of a regulated market. Hence, we may expect that this data is the “best” that is available and that the results are poorer and risks are higher in settings where the lack of regulation leaves more room for exploitation. Within these limitations, it has been shown that kidney vendors are subject to greater clinical risks as compared to other voluntary living donors within the same country [75–78].

The differences in quality of life and psychological impact are even more striking: even if voluntary kidney donation may not always be glittering gold, the psychological experiences of kidney vendors are highly negative, in particular when compared to the experiences of voluntary kidney donors [78–81].

## Justice

There are several legitimate ways to consider justice: justice as the fair distribution of opportunities and resources; justice as a moral – ethical right, considering the different religious points of view; justice as laws.

### Justice as a fair distribution of opportunities (recipient and donor)

The idea is that the burdens and benefits of treatments should be distributed fairly among all groups in society. In this regard, justice may be evaluated according to four main areas: fair distribution (crucial in the case of scarce resources), competing needs, rights and obligations, and potential conflicts with the established legislation [82–86].

Once again, the distribution may be interpreted in different ways, for example distribution of financial resources, of health care access, as well as of the treatments themselves, as in the case of kidney transplantation which is financially advantageous as compared to dialysis, but kidneys from deceased donors are not available for every patient, or at least are not available within a short period of time [87–91].

Therefore, this concept of justice may be interpreted differently: if justice is meant as an overall fair distribution, then there are striking differences between donor and recipient, or in other words seller and buyer: here the question of the obvious inequality merges into the vast problem of poverty, leading to further exploitation of the poor. No doubt there is a strong conflict of interest between the donor and the recipient, and the need to sell a kidney in itself suggest unequal access to the basic needs of society, including health care.

Conversely, if the problem of justice is meant in the sense of optimizing the limited resources of transplantation, then the choice may not be unsound: the patient is not in competition with other subjects for the limited number of grafts available from deceased kidney donors and, on a larger scale, since dialysis costs more than transplantation, this choice may also be favorable to his/her own health care system, at least in cases such as this one in which the donor and the recipient do not reside in the same country. But, if we shift the scenario to a global level, we have to consider the possibility that the clinical harm to the donor hinders the advantages for the recipient [46, 47, 78, 79, 92–94].

The role of the physician is crucial: is he/she the advocate of his/her patient and/or of his/her society, or is he/she a global advocate of all patients in defense of human rights?

Hence, the controversy regarding the patient’s position inevitably merges with the enormous problem of defining the role of the physician in the present globalized society: is the physician a skilled technician, a detached counselor or a moral agent? [95–97].

### Justice as moral understanding or as a religious obligation (both parties)

While an extensive analysis on the different religious attitudes towards organ trade is far beyond the scope of this review, a simplification of this hyper-complex subject may be a Manichean division between ethical-religious positions, substantially banning organ trade, and a pragmatic position, favoring the short term advantage for all parties (money for the donor, kidney for the recipient).

Moral justice is not the same as legal justice: while in an ideal world ethical principles, moral understanding and legal positions should probably merge, this is not an ideal world, and the laws may be different thereby reflecting various pragmatic, religious or social backgrounds and choices as well [98–105].

The codes regulating the medical profession lie somewhere between an ethical-moral code and the law: in fact, at least in some countries, in the absence of a law an ethical code may take on the same importance as the law. This was the case with the Nuremberg code in Europe, but it is not the case with the Istanbul declaration, that however is forcing governments to define a clear position towards organ trafficking (as recently occurred in China), and is finally trying to stop the sale of human organs [17, 36–38, 106].

Before the Declaration of Istanbul in 2008, similar positions had already been taken, for example by the World Health Organization: “The Guiding Principles on Human Organ Transplantation” (1991) banning the commercialization of human organs as 'a violation of human rights and human dignity', and by the European Convention on Human Rights and Biomedicine Concerning Transplantation of Organs and Tissues of Human Origin (2002), condemning organ and tissue trafficking, calling on States to provide appropriate sanctions [107, 108].

The Istanbul declaration presents two further elements of great interest and novelty: first of all, it provides a clear definition of organ trafficking, transplant commercialization, and transplant tourism: “Travel for transplantation is the movement of organs, donors, recipients, or transplant professionals across jurisdictional borders for transplantation purposes. Travel for transplantation becomes transplant tourism if it involves organ trafficking and/or transplant commercialism or if the resources (organs, professionals, and transplant centers) devoted to providing transplants to patients from outside a country undermine the country’s ability to provide transplant services for its own population” [106].

Secondly, for the first time in the history of Nephrology, the Istanbul declaration- choose presents a medical association (the ISN) as the promoter of a shared legal position, thus interpreting the medical profession not only as a technical one but also as a moral and social one [106].

### Justice as law

Organ trafficking has been defined as a crime that occurs among vulnerable categories of people. However, the legal definition of a crime is different from the moral definition of a crime and several positions are present worldwide: organ commerce may be regulated and/or tolerated in the absence of a law, or banned by a law.

This latter position may also have different aspects: in most of the countries where this study was undertaken, such as Italy, that ban the organ trade, there is no mention of the treatment of patients who bought a kidney in a foreign country where this practice may be allowed, regulated or simply tolerated. Hence, as in the example discussed herein, the patient may receive all the care he/she needs, regardless of the origin of his/her kidney.

In some countries, such as Germany, buying an organ is considered a crime and the recipient is prosecuted when he/she returns to his/her homeland [109]. Both positions have pros and cons: the first may reflect greater attention to privacy and the respect of a basic tenet of bioethics, that is, caring without judging; however, such an attitude implicitly encourages exploitation in poorer countries (and may even be beneficial for the health care system, as previously discussed) [110, 111]. The second position is probably more effective in preventing organ trade, but once more affects the physician’s role making him/her a controller of social rights, and a guardian of the laws [112, 113].

### Autonomy: first actor, the recipient, the buyer

The Principle of Respect for Autonomy states that the patient's preferences have to be respected as long as the patient is informed of the benefits and risks, has understood this information, and has given consent [114–116].

Once more, with subjects who seek medical advice, like our patient, the discussion shifts to the information and to its modality, in other term, to the physician/patient relationship. It also shifts to the different boundaries surrounding the transmission of the information, i.e., according to the relationship. In a paternalistic relationship the physician would try to convince the patient of the “right” choice, while following a technical approach the expert would merely confront the patient with risks, advantages and uncertainties, and through a therapeutic alliance both parties would be expected to openly discuss their opinions and try to find a shared clinical pathway [117–120].

### Autonomy: second actor, the donor, the seller

This is probably the most crucial point that divides the advocates of the kidney trade from those who would ban it [121–125]. Once more there are several levels of discussion. The first one is quite simple: does a very poor person, i.e., one who is so poor as to decide to sell one of his/her body parts, truly have “free power of choice, without the intervention of any element of force, fraud, deceit, duress, over-reaching, or other ulterior form of constraint or coercion”, to again cite the Nuremberg declaration?

However, does the speculative decision to consider the potential seller (who is not free in his/her choice) actually deprive this person of the only chance to improve his/her quality of life and/or to avoid personal dramas?

Once more, the answer implies the decision of a speculative-theoretical-philosophical position versus a pragmatic one, inevitably shifting the discussion to a different level: does being a physician imply the choice of an ethical position or is the physician a pragmatic actor in a wider world? These positions further merge into the concept of the human body i.e., as a whole, as a series of pieces, as an individual good or the as the property of a society? [28, 126, 127].

As recently underlined by a series of focus groups in European countries, people with similar cultural and religious backgrounds may respond differently to questions on human organs, spanning from “I’m mine” to “the body is not a car”, thus underlining, above all, the limits and risks of oversimplification [28, 128].

### A role for narrative ethics?

While principlist ethics may offer a valuable frame for dissecting the clinical problem and for analyzing the main issues, a narrative approach may be more apt to identify solutions in individual cases, taking into account the patient’s history, the presence of family support, the presence of fears and concerns, as well as his/her daily life and job [129–131].

It has been said that “a narrativist tries to capture the stories” that patients and families tell about how they came to be in a particular predicament as well as what was behind their moral decision-making at earlier important moments [129]. The flexibility of narrativism may compensate for the more rigid structure of the four principles.

The history of our patient underlines the capital importance of his fears of being sick that led him to initially deny his disease and then to avoid follow-up thus leading to negative consequences. However, when compelled by his clinical conditions to start a diet and to undergo medical treatment, he was able to follow the therapies with good compliance, also thanks to the presence of strong family support.

In spite of his (apparent) determination to buy a kidney, he had asked his physician for advice, suggesting that his convictions were probably weaker than he claimed, implicitly asking for help and leaving room for discussion. These considerations were at the basis of an attempt to dissect the patient’s fears and expectations as a guide to an empirical counseling process that also involved his family (see epilogue).

Once again, attention shifts from the role of the physician as a technical albeit empathic counselor to the physician as a moral agent, a role defined as “capable of acting with reference to right and wrong”, and who is therefore responsible for his-her decisions [97, 132, 133]. In this regard, principlism and narrative approaches have the role to support the physician in better understanding the ethical problems, enabling a “right decision”. According to this approach, that stresses physician’s responsibility, intellectual and moral virtues are needed aside clinical knowledge in the resolution of specific dilemmas [132, 133].

### Case epilogue

During the first talk, the nephrologist mainly listened to the patient and explained to him that in spite of the personal opinions against organ trade shared by most of the International Nephrology community, the physician is not a judge, the problem of organ trade is still matter of debate, and as a counselor, he intended to maintain the right to express his personal opinions.

The physician also clarified that in any case, according to the local laws, if the patient had chosen to buy a kidney the physician would be able to take care of him after kidney transplantation and, after having pointed out that transplantation may require long hospitalization and that it bears surgical and clinical risks, he asked the patient to come back with his family for further discussion.

The physician also asked the patient to have a talk with a psychologist as his choice appeared to be driven by fear and anguish, and he also gave him a paper to read on this issue (Kidney vending: "Yes!" or "No!" [121]) pointing out that this paper did not take into account the presence of poverty, or criminality but was intended to give some insights into the complexity of what, at that moment, seemed to the patient to be a “reasonable choice”.

The paper reported a somewhat similar case, and although the report was relatively old, the physician decided that it could be a good starting point for an analysis of the problem without forcing the patient to face the toughest aspects of commercialism (exploitation, poverty etc.) right away in the first step [121].

The whole family participated in the following meeting. The discussion followed the four basic principles, and they discussed in detail the expected benefit and the “non- “non detriment”, and the disadvantages likely to be experienced by the vendor, according to the international literature that was supplied to the patient and his family. The patient was also informed that he should analyze the legal aspects, if necessary with a legal expert, if he decided to go forward with his choice.

After having asked for several clarifications on life on dialysis, and having accepted to meet a couple of young home hemodialysis patients, the patient and his family decided to reflect more at length on the moral and religious aspects of the problem, with the support of a catholic priest who had an important role in the family.

A few weeks later, the patient reported that he had decided to give up his plan, and that both he and his wife had decided to undertake psychological support. He started dialysis 4 months later while he was later placed on a waiting-list for a deceased donor kidney transplantation. He died of sudden death 6 months after the start of dialysis.

The family remained on good terms with the physician, and the wife once said “ we are grateful that we did not experience the sorrow of a sudden death abroad after a kidney transplant carried out against his own ethical principles”.

## Conclusions

This is not a happy ending to the story: our patient died while waiting for a kidney transplant. However, the family did not perceive his death as avoidable by a timely graft, and considered that, as grim as it was, the outcome was not linked to a choice that conflicted with moral and ethical rules that, in a moment of anguish, risked being ignored.

From a clinical point of view, sudden death is more common in dialysis patients; a possibility exists therefore, that had he undergone early transplantation his outcome would have been different, taking into account that the patient was considered a good candidate for kidney transplantation. The family never asked if a timely graft would have changed the outcome, and this point was never touched on in the several further conversations.

Three major points of the decisional pathway should be be underlined: the usefulness of breaking down the problems according to the “major” principles; the importance of the patient-physician relationship also as a pathway to undertake a narrative, personalized approach, and the step-by-step follow-up of the decision, involving the patient and his/her family in a discussion grounded on the available evidence.

Single cases are not general laws, and several questions remain open, such as the “best” degree of the physician’s involvement in the discussion; the importance of third party guidance, in this case the catholic priest; the level of the information to be supplied, and the pathway of information (a “softer pathway” as was the one that was chosen, or a “harder one” presenting the evidence on exploitation first).

Narrative ethics places the decisions in the physician’s hands, leaving him/her to discuss (possibly with the help of other experts) each issue adapted to what is considered “best for the single patient”, according to a decisional process, that corresponds to that of “personalized medicine” which is increasingly being identified as the best option for patients affected by chronic diseases.

Unlike with clinical medicine, that strives to define the single best solution, an ethical discussion should first of all lead to the understanding that there is no single best solution, in particular when the four principles show multiple, critical conflicting points as is the case herein.
